# Supplementary figures and images for: Experimental co-transmission of Simian Immunodeficiency Virus (SIV) and the macaque homologs of the Kaposi Sarcoma-Associated Herpesvirus (KSHV) and Epstein-Barr Virus (EBV)
Source: PLoS One. 2018 Nov 16;13(11):e0205632. doi: 10.1371/journal.pone.0205632 (PMC6239284; doi:10.1371/journal.pone.0205632)

S1 Fig. RV1 qPCR assay with comparison to sequenced PCR products

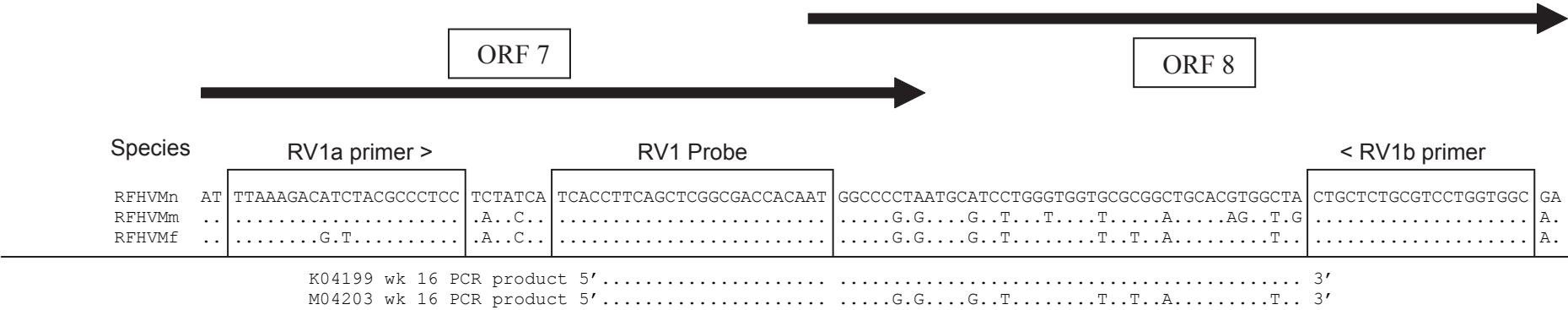

Supplement: S1 Fig — (PDF) [file pone.0205632.s001.pdf]
